# The effect of patient anxiety and depression on motion during myocardial perfusion SPECT imaging

**DOI:** 10.1186/s12880-016-0153-9

**Published:** 2016-08-22

**Authors:** Vassiliki Lyra, Maria Kallergi, Emmanouil Rizos, Georgios Lamprakopoulos, Sofia N. Chatziioannou

**Affiliations:** 1 2nd Department of Radiology, Nuclear Medicine Section, National and Kapodistrian University of Athens, Attikon Hospital, 1 Rimini St., Athens, 12462 Greece; 2 Department of Medical Instruments Technology, Technological Educational Institution of Athens, TEI, 28 Ag. Spiridona St., Athens, 12210 Greece; 3 2nd Department of Psychiatry, National and Kapodistrian University of Athens, Attikon Hospital, 1 Rimini St., Athens, 12462 Greece; 4 Nuclear Medicine Section, Biomedical Research Foundation Academy of Athens, BRFAA, 4 Soranou Efesiou St., Athens, 11527 Greece

**Keywords:** Patient motion, Anxiety, Myocardial perfusion SPECT imaging, Artifacts, Depression

## Abstract

**Background:**

Patient motion during myocardial perfusion SPECT imaging (MPI) may be triggered by a patient’s physical and/or psychological discomfort. The aim of this study was to investigate the impact of state anxiety (patient’s reaction to exam-related stress), trait anxiety (patient’s personality characteristic) and depression on patient motion during MPI.

**Methods:**

All patients that underwent MPI in our department in a six-month period were prospectively enrolled. One hundred eighty-three patients (45 females; 138 males) filled in the State-Trait Anxiety Inventory (STAI) and the Beck Depression Inventory (BDI), along with a short questionnaire regarding their age, height and weight, level of education in years, occupation, and marital status. Cardiovascular and other co-morbidity factors were also evaluated. Through inspection of raw data on cinematic display, the presence or absence of patient motion was registered and classified into mild, moderate and severe, for both phases involved in image acquisition.

**Results:**

The correlation of patient motion in the stress and delay phases of MPI and each of the other variables was investigated and the corresponding Pearson’s coefficients of association were calculated. The anxiety-motion *(r = 0.43, P < 0.0001)* and depression-motion *(r = 0.32, P < 0.0001)* correlation results were moderately strong and statistically significant for the female but not the male patients. All the other variables did not demonstrate any association with motion in MPI, except a weak correlation between age and motion in females *(r = 0.23, P < 0.001)*.

**Conclusions:**

The relationship between anxiety-motion and depression-motion identified in female patients represents the first supporting evidence of psychological discomfort as predisposing factor for patient motion during MPI.

## Background

Good quality data is crucial in order to achieve high diagnostic accuracy in myocardial perfusion SPECT imaging (MPI) [1–5]. Prior to image processing, raw data should be reviewed for image quality and patient motion. Patient motion is considered the most frequent cause of artefactual defects resulting primarily in false positive results [1, 3].

Patient motion has been simulated and evaluated in a variety of studies using different methods [4–9]. Results have not demonstrated a direct correlation between motion pattern (type and magnitude of motion) and imaging outcome [8]. Different semi-automatic and automatic “motion correction” software has been developed to identify and analyze the patient’s (voluntary and involuntary) motion, in order to realign the projection data before image reconstruction [10–16]. However, none of the available software is considered accurate enough to capture both the variety and complexity of patient motion, and as a result, the reconstructed image should be interpreted with caution [16]. Therefore, the best approach regarding patient motion is prevention. “Heart motion”, due to involuntary motion, such as “Respiratory motion” [17, 18] and “Upward creep” [19, 20] could potentially be prevented through delay in the initiation of acquisition. In contrast, “heart motion”, due to voluntary body movements, could be minimized by preventing patient’s discomfort [3, 21]. For this reason, the patient should always be in a comfortable position during imaging [21]. Lumbar and knee supporters could minimize back strain caused by the patient being on supine position with overextended left arm. In addition, the patient should always be informed of the negative effects of motion on diagnosis. Despite these routinely taken precautions, motion problems are not alleviated.

The patient’s anxiety has been considered another cause of the patient’s psychological and physical discomfort and thus contributing to patient’s motion during MPI and other imaging modalities [3]. Anxiety is described as a state of emotional distress and inner turmoil, which may be manifested by nervous behavior and restlessness, muscular tension and other somatic complaints [22–24]. Anxiety-related sympathicotonic and claustrophobic reactions and some anxiety reduction protocols have been evaluated mainly for magnetic resonance imaging examinations [25–30]. The aim of this study is to investigate the impact of *state anxiety* (patient’s reaction to exam-related stress), *trait anxiety* (patient’s personality characteristic), and *depression* on direct patient motion during myocardial perfusion imaging.

## Methods

### Patients

**Fig. 1:**
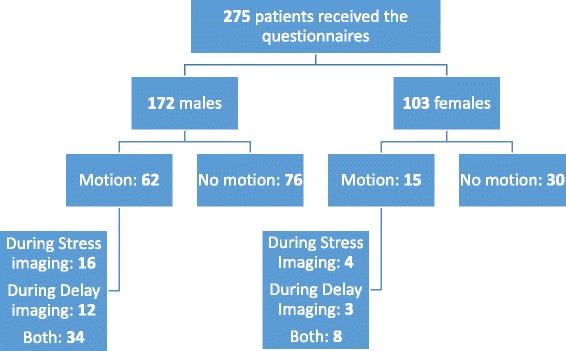
Patients’ flow chart

Two psychometric instruments were administered to 275 consecutive patients (172 men, 103 women) that underwent MPI in our department within a six-month period. The patients were required to fill-in two questionnaires; the one addressed the level of state and trait anxiety of the patients prior to the procedure and the other the level of depression. Forty-nine patients (17.8 %) did not complete the questionnaires while 43 patients (15.6 %) completed them partially. It is noteworthy that the majority (63 %) of these 92 patients were women, while the majority (62.5 %) of patients who were asked to fill-in the questionnaires were men. All of these 92 patients were excluded from the study (Fig. 1).

One hundred and eighty-three (183) patients, 45 women and 138 men, accurately completed and signed the two questionnaires and their responses were evaluated in this study. Demographic information including age, years of education, occupation, and marital status, and anthropometric variables (weight and height) were also gathered. The body mass index (BMI) was calculated according to the relationship: BMI = weight in Kg/(height in m)^2^. The difference in sample sizes between male and female patients was due to a) the smaller proportion of women who underwent MPI (37.5 %, i.e. 103 out of 275 patients), and b) the higher proportion of women (56.3 %, 58/103) compared to men (19.7 %, 34/172), who either refused to complete the questionnaires or completed them partially.

In preparation for the MPI test, information about the patient’s cardiovascular and medical history was gathered. Variables evaluated were the presence of known or suspected coronary artery disease, the presence of left ventricular systolic dysfunction (ejection fraction (EF) ≥50 % or EF < 50 %), as well as other comorbidity factors, such as cancer, serious hematologic disorders, hemodialysis, cerebrovascular disease, complicated long standing diabetes mellitus, severe connective tissue disorders, etc.

### Psychometric questionnaires

Patients were provided with the first questionnaire and were informed about the succeeding second one, by two physicians of our department, upon arrival. Both physicians had quite similar approach to the patients, in order to minimize interferences.

The *Spielberger’s State-Trait Anxiety Inventory* (STAI) [31, 32], is a two-page questionnaire based on a 4-point Likert scale consisting of 40 simple questions on a self-report basis. Theoretically, a 6^th^ grade graduate could readily comprehend and complete them in about 10 min. The STAI measures two types of anxiety: the state anxiety or temporary anxiety about an event, and the trait anxiety or daily anxiety level as a personal characteristic [31, 32]. Namely, the part of state anxiety inventory measures the extent of heightened emotions and overreaction to a situation- a perceived stressor- such as undergoing an MPI imaging. Everyone experience some anxiety, something similar to fear, which is out of proportion to the actual risk involved. The common symptoms of anxiety and fear may include uneasiness, spells of perspiration, bouts of frequent urination and muscular tension, whereas all symptoms can be associated with the release of specific neurohormonal mediators [22, 23].

The part of trait anxiety inventory measures the level of “neuroticism” and unfocused worry which stems from the personality of an individual and induces his stable tendency to respond with anxiety in the anticipation of situations [23, 24]. Higher scores on STAI questionnaire are positively correlated with higher levels of anxiety [31, 32]. Normal values for state anxiety in the Spielberger’s healthy population aged 30–59 years, was 38.1 ± 10.1 for females and 37.3 ± 9.8 for males [31]. This questionnaire had been adapted by A. Liakos and S. Giannitsis in the 1980s to suit the Greek population [33], and since, its validity had been re-evaluated by using a random sample of Greek university students and civil servants, with a mean age of 31.71, consisting of 64.8 % females. Mean anxiety state score was 43.21, mean anxiety trait score 42.79 and the resulting STAI score was 86.1 [34].

To maximize time management, the patients were provided with the second questionnaire, known as *Beck Depression Inventory* (BDI) [35, 36], while waiting for the delay phase of MPI. BDI is a commonly used instrument for quantifying levels of depression. It contains a 21 item self-report, each with four possible choices scored on a scale of 0 to 3. Higher total scores indicate more severe depressive symptoms. The standard cutoffs used are [35, 36]: 0–13: minimal depression; 14–19: mild depression; 20–28: moderate depression; and 29–63: severe depression.

### Image acquisition and processing

All patients underwent ergometric (treadmill) or pharmacological (adenosine or dipyridamole) stress. SPECT imaging was performed at 10 min (stress) and 4 h (delay) following intravenous injection of 3 mCi Tl-201. A single-head GE Millennium camera, with a LEGP collimator was used for all cases. Energy window was set at 20 % at 72KeV and 30 % at 167KeV. 32 views of 40 s each, stored in a 64x64 matrix, were acquired for a 180° orbit and a total acquisition time of ~22 min. The pixel size was 6.4 mm. The raw re-projection data was reconstructed by using a filtered backprojection (FBP) algorithm (Butterworth; cutoff frequency, 0.39; power, 10) and an Ordered-Subset Expectation Maximization (OSEM) iterative reconstruction algorithm, on the Xeleris workstation (GE Healthcare).

### Detection of patient motion

The analysis of images for patient’s motion was based on inspection of the raw data on cinematic display, which was performed by an expert nuclear physician. The absence of motion had to be confirmed on the sinogram image as well [10]. However, motion was regarded as present only when the observer was certain of its existence. Each study was assessed for the presence of visually detectable motion, the type of motion and the grade of motion. The types of patient motion were classified as suggested in the literature [3, 5] in a) bounces (brief up or down movements, observed in <3 frames before returning to the original y-coordinate), b) shifts (up or down movement involving all the remaining frames), c) complex motion (multiple bounces or combination of bounces and shifts), d) lateral movements and translations (rotations of the body around its axis). Patient motion was scored on a scale from 1–3 as *absent* (0), *mild* (1), *moderate* (2) and *severe* (3) depending on the degree and recurrence of motion during rotating cinematic display of raw data. A single subtle event of motion throughout the dataset was identified as mild, while several single events (complex motion) were characterized as severe motion. In case of ambiguity about the largeness of a motion event, distance between lower edge of the image and lowest part of the heart silhouette was compared on the selected frames. Single events of motion, higher than 3 pixels, were characterized as severe. The aforementioned assessment of patient motion was scored for the two sets of raw data, for those of the stress phase called “*Patient Motion Stress*” and for those of the delay phase called “*Patient Motion Delay*” (Fig. 1).

The visual inspection and assessment of motion by one expert was evaluated by repeating the process almost one year after the previous one. In case of a major discrepancy, compared to the previous patient motion evaluation results (patient motion absent instead of present or vice-versa, or a motion score difference of more than 2 points regarding a specific stress or delay phase), the decision was made in cooperation with another nuclear physician expert.

**Fig. 2:**
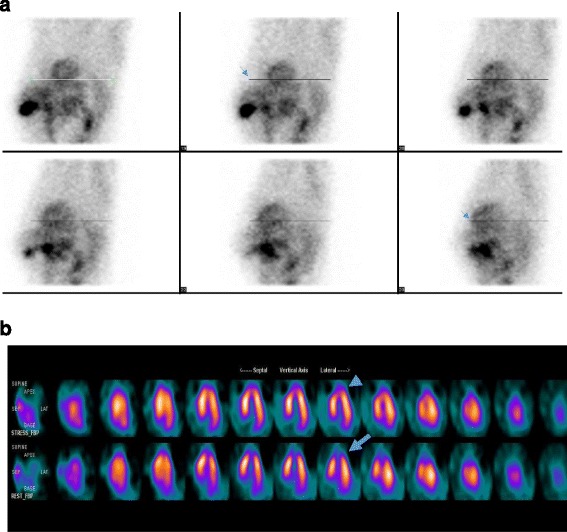
**a** From inspection of the raw data (32 planar images) in cinematic display, which were acquired during the “delay phase” of MPI, a downward patient movement was detected. Using tracing lines at the edges of heart silhouette on each of the 32 planar views, the patient’s displacement (shift) was confirmed on images 19 to 23 (*arrows*). This is a “Patient Motion Delay” assessment classified as grade 2 (moderately severe motion). **b** The tomographic filtered back projection (FBP) reconstruction of the raw data illustrated on Fig. 2a, revealed a large myocardial apical defect perfusion, which was diagnosed as motion artifact (*arrow*). Conversely, no myocardial apical defect appeared after the tomographic reconstruction of the “stress phase” images (*arrowhead*), since the patient remained motionless during the data acquisition

A representative case of “Patient Motion” evaluation and of the corresponding “Motion Artifact” are demonstrated in Fig. 2a and b respectively.

### Statistical analysis

Our final database included both qualitative (ordered and unordered) and quantitative (discrete and continuous) types of data. Qualitative variables included *gender*, *occupation*, *marital status*, *major comorbidity*, *SPECT evaluation*, *cardiac disease class*, *patient motion stress*, and *patient motion delay*. Quantitative variables included *education*, *age*, *weight*, *height*, *BMI*, *time of cardiac disease diagnosis*, *state anxiety score*, *trait anxiety score*, *STAI score* (sum of the two anxiety scores), and *BDI score*. The characteristics and the descriptive statistics (mean values and standard deviations) of the study are listed in Table 1.

**Table 1:** Characteristics of the participants in the MPI motion study

Characteristics of patients	Total patients^a^ (183)	Male patients^a^ (138)	Female patients^a^ (45)
Age (years)	63.3 ± 10.2	62.7 ± 10.1	65.0 ± 10.6
Education (years)	10.7 ± 3.0	11.1 ± 9.2	9.4 ± 2.7
BMI	28.7 ± 4.8	29.1 ± 4.8	27.7 ± 4.9
Occupation (%)			
0 = Homemaker	25 ± 14	0 ± 0	25 ± 56
1 = Retired	89 ± 49	75 ± 54	14 ± 31
2 = Employed	57 ± 31	53 ± 38	4 ± 9
3 = Unemployed	12 ± 7	10 ± 7	2 ± 4
Marital status (%)			
0 = Single	9 ± 5	8 ± 6	1 ± 2
1 = Married	148 ± 81	115 ± 83	33 ± 73
2 = Widow/er	11 ± 6	3 ± 2	8 ± 18
3 = Divorced	15 ± 8	12 ± 9	3 ± 7
Major comorbidity (%)			
0 = No	158 ± 86	120 ± 87	38 ± 84
1 = Yes	25 ± 14	18 ± 13	7 ± 16
Cardiac disease (%)			
0 = Negative	76 ± 42	45 ± 33	31 ± 69
1 = EF ≥50 %	84 ± 46	72 ± 52	12 ± 27
2 = EF < 50 %	23 ± 13	21 ± 15	2 ± 4
Time of cardiac disease diagnosis (years)	2.7 ± 4.3	3.1 ± 4.5	1.5 ± 3.3
SPECT evaluation			
0 = Normal	89 ± 49	53 ± 38	36 ± 80
1 = Mildly abnormal	43 ± 23	38 ± 28	5 ± 11
2 = Moderately abnormal	34 ± 19	30 ± 22	4 ± 9
3 = Severely abnormal	17 ± 9	17 ± 12	0 ± 0
Anxiety state score	41 ± 13.2	38.9 ± 12.9	47.5 ± 12.1
Anxiety trait score	44 ± 9.4	42.5 ± 8.9	48.7 ± 9.4
STAI score	85 ± 21.1	81.3 ± 20.1	96.3 ± 20.3
BDI score	13.6 ± 9.1	12.2 ± 8.0	17.9 ± 10.8
Patient motion stress			
0 = Absent	123 ± 67	90 ± 65	33 ± 73
1 = Mild	20 ± 11	15 ± 11	5 ± 11
2 = Moderate	25 ± 14	19 ± 14	6 ± 13
3 = Severe	15 ± 8	14 ± 10	1 ± 2
Patient motion delay			
0 = Absent	126 ± 69	92 ± 67	34 ± 76
1 = Mild	25 ± 14	19 ± 14	6 ± 13
2 = Moderate	17 ± 9	14 ± 10	3 ± 7
3 = Severe	15 ± 8	13 ± 9	2 ± 4

^a^mean values ± standard deviations

Our primary interest was the investigation of possible correlations between patient mood and patient motion in the stress and delay phases of MPI. Hence, the Pearson’s coefficients of association were determined for pairs of variables.

Associations between categorical variables were also studied by generating 2x2 contingency tables and applying Fisher’s two-tailed exact test. The level of significance for all tests was set to 5 %. In the investigation of possible associations, linear or non, between pairs of the variables listed in Table 1, the two variables of motion (Patient Motion Stress & Patient Motion Delay) were considered as *responses* or dependent variables, while all others were considered as *predictors* or independent variables.

## Results

Patients with a basic level of education (22 males, 13 females) required approximately two to three times more than the expected 10 min to complete the STAI and even longer, exceeding half an hour, to complete the BDI questionnaire. The patient motion evaluation had excellent intra-observer agreement (96.1 %, 176/183 patients).

The primary hypothesis of this study was that the patient’s pre-scan psychological state, was associated with patient motion during MPI. Hence, the corresponding pairs of variables were studied for possible associations. Table 2 shows the results of the Pearson’s coefficient and its *P*-value for the variables listed in Table 1 for all, male and female patients. The results in Table 2 indicate that there is a strong, statistically significant correlation between patient motion at stress phase and patient motion at delay phase of MPI. There is only a weak but statistically significant association between trait anxiety and patient motion in the delay phase of MPI and between the STAI score and motion in the same phase. With the exception of the motion during the two phases of imaging, only 2-3 % of the variance in one of the variables is accounted for by the variance in the other variable.

**Table 2:** Pearson’s correlation coefficient, coefficient of determination, and P-values for selected pairs of variables for all 183 patients and for the 138 males and the 45 females separately

Variables	Pearson’s coefficient (*r*)			Coefficient of determination (*r* ^*2*^)			*P-*value		
	All	Males	Females	All	Males	Females	All	Males	Females
Motion S**/**Motion D	0.66	0.64	0.73	0.43	0.41	0.53	<0.0001	<0.0001	<0.0001
State-A**/**Motion S	0.12	0.12	0.31	0.01	0.01	0.10	0.12	0.12	<0.0001
State-A**/**Motion D	0.14	0.12	0.42	0.02	0.02	0.17	0.05	0.10	<0.0001
Trait-A**/**Motion S	0.14	0.14	0.34	0.02	0.02	0.12	0.06	0.07	<0.0001
Trait-A**/**Motion D	0.16	0.15	0.38	0.03	0.02	0.15	0.03	0.05	<0.0001
STAI**/**Motion S	0.13	0.14	0.34	0.02	0.02	0.12	0.07	0.07	<0.0001
STAI**/**Motion D	0.16	0.15	0.43	0.03	0.02	0.18	0.03	0.05	<0.0001
BDI**/**Motion S	0.04	−0.01	0.32	0.00	0.00	0.10	0.55	0.98	<0.0001
BDI**/**Motion D	0.07	0.05	0.28	0.01	0.00	0.08	0.32	0.51	<0.0001

Motion S = “Patient Motion Stress”, Motion D = “Patient Motion Delay”, State-A = “State Anxiety score”, Trait-A = “Trait Anxiety score”

Moreover, anxiety was significantly positively correlated with depression both in males (*r = 0.56; P < 0.001*) and females (*r = 0.67; P < 0.001*) but the women of the study had significantly higher anxiety and depression scores compared to men when taking the MPI test (Table 3). The *two-tailed P-value* for the data in Table 3 is *0.0096*, indicating that the association between the groups (male, female) and the outcomes (trait anxiety scores) are statistically very significant. A similar result was found for the anxiety state scores (*P = 0.0004*), the STAI score (*P = 0.0011*) and the BDI score (*P = 0.0028*).

**Table 3:** 2x2 contingency table for the male and female patients and the trait anxiety assuming the mean score (44) of the entire study population as cutoff point

	Outcome		
Group	Trait-A < 44	Trait-A ≥ 44	Totals
Male	75	63	138
Female	14	31	45
Totals	89	94	183

Trait-A = “Trait Anxiety score”

The most interesting result was that the association between anxiety or depression and motion was statistically significant (*P = 0.0001*) for women but insignificant for men. A higher anxiety STAI score in women was correlated with a moderate risk of motion and this correlation was more evident in the delay phase of MPI *(r = 0.43)*. Similarly, a higher BDI score was correlated with a mild to moderate risk of motion (*r = 0.32)* (Table 2). In contrast to the abovementioned observation, a smaller proportion of women moved during image acquisition (33 %, 15/45 females) compared to men (45 %, 62/138 males) while their motion was less severe (13 %, only 2/15 females had a motion score ≥ 3) than that of men (29 %, 18/62 males had a motion score ≥ 3). This is in accordance with what has been described in a previous study [15]. According to Table 4, this difference is not statistically significant for our study, most likely due to population differences (138/183 patients are men, which is 75 % of the study population). The *two-tailed P-value* for the data in Table 4 is *0.3636* and the association between the two groups of patients and the motion outcome was found not to be statistically significant. Similar results were obtained for the delay phase study (*P = 0.3541*). Finally, to account for population size difference, we matched 42 male to 42 female patients in terms of demographic and physical characteristics. Results were similar for the matched groups, i.e., no statistically significant associations.

**Table 4:** 2x2 contingency table for the male and female patients and motion outcome in the stress phase of MPI

Group	Outcome		
	No motion	Motion	Totals
Male	90	48	138
Female	33	12	45
Totals	123	60	183

Patients with motion ratings 1–3 were grouped together under the “Motion” outcome

Other variables, such as age, education, BMI, occupation, marital status, comorbidity, time and severity of heart disease, were investigated for possible associations with the motion in MPI. Age of the female population of the study was weakly correlated to the motion in the stress phase of imaging (*r = 0.23; P < 0.001*). Secondly, these variables were also examined for potential associations with the anxiety and depression results. Only married female patients (73.3 %, 33/45) had a weak but statistically significant correlation to the trait anxiety of these patients (*r = 0.16; P = 0.03*). All other associations were not statistically significant.

## Discussion

The association of higher state-anxiety scores in patients undergoing MPI, in both males and females has been proved in a previous study [37]. For the first time, to our knowledge, our study investigated the effect of anxiety and depression in patient motion during MPI, whereas common psychometric STAI and BDI questionnaires were used for the assessment. Our hypothesis was that both state and trait anxiety were associated with patient motion during MPI, assuming that patients with state and trait anxiety moved more during imaging than patients with depression. Hence, according to a simplified definition of abovementioned psychological disorders, we hypothesized that anxiety patients who experience tension and uneasiness more frequently [23] should have a higher risk of motion, while depressive patients who more frequently experience fatigue and stillness [38], should have a smaller risk. Finally, since anxiety disorders and depression may co-exist [39–41], only the prevalent condition should affect the patient’s motion during data acquisition.

Interestingly, our results in respect to anxiety related motion and depression related motion were statistically significant only in women *(P = 0.0001*) but not in men. A higher anxiety STAI score was moderately correlated to motion. Contrary to our hypothesis, even a high depression BDI score was marginally, but still positively, associated to motion in women (Table 2). Although stillness and immobility are characteristics of melancholic (typical) depressive syndromes, it is also true, that many depressive patients (atypical depression) experience a feeling of uneasiness and are unable to stay at rest [38, 41], similarly to anxiety disorders.

The significant association of anxiety and depression scores [39–41] and, moreover, the significantly higher anxiety and depression scores in women compared to those in men, observed in our study (Table 3), are in line with the existing literature [31, 42–44]. In general, women present higher rates of anxiety and depression, and the higher rate of diagnosis (more likely report their symptoms and ask medical support) [42, 43] and the different biological (neurohormonal) [45] and social background (e.g. caregiving) [43, 46] have been involved, although the combination of these factors into integrated aetiological models continues to be lacking [47].

In an MRI study [48], the relationship between clinically relevant motion artifacts and pre-scan state anxiety scores was examined. There was no statistical significant relationship (*P = 0.30*) between state anxiety scores and motion artifacts in both men and women, something that is not in line with our results in women. That may be due to the fact, that the end points in both studies were different, since the overall motion was evaluated (75/183 moved) in the present study, while only the motion artifacts were evaluated (19/278 had motion artifacts) in the MRI study.

The presence of less motion in women, as was our initial impression, as demonstrated by a previous study [15], should not appear contradicting to the abovementioned result of a high anxiety score and its positive correlation with motion in women. This actually means, that anxiety and depression do influence the motion of female patients during MPI but do not explain the motion in men. The relative higher rate and higher score of motion in men is an independent variable. None of the other parameters examined, were found to justify motion in men.

Indeed, the anthropometric, demographic and medical information was collected, in our effort to investigate a wider range of variables correlated to the motion or the anxiety and depression scores, in order to provide a better distinction of patients at greater risk for motion.

The occupation and the marital status have been involved to both anxiety and depression, in literature [49]. Married women showed a weak but statistically significant association to the trait anxiety score, which is in accordance to some specialized literature, stating that married women could be more stressed compared to singles [44]. Twenty-two married women in our study, almost half (48.8 %) of our study’s population, were elderly housewives or retired, who underwent MPI in order to evaluate an atypical chest pain. They all had a normal MPI. Anxiety could be associated with high rates of medically unexplained symptoms and some women, even without a professional activity, may experience a greater stress burden due to an important care-giving role to their grandchildren or other family members [46, 50].

Furthermore, an underlying pathology, such as heart failure, may precipitate anxiety and depression, both recently defined as “inflammatory” mood disorders [51–53]. However, our results in respect of heart failure related to motion and of heart failure related to anxiety and depression scores were not statistically significant in men, (almost all our heart failure cases involved men).

A previous study [16] showed that there was no statistically significant association between age and motion. Even though increased age and BMI are factors generally of lack of physical fitness and therefore elementary predisposing factors of patient discomfort which could be aggravated during imaging. However, our study did not demonstrate an overall association between age and motion, too, except for older women who were found to move slightly more. BMI was not found to have an association to patient motion in either men or women.

The strong linear correlation between patient motion at stress and patient motion at delay, confirmed our suspicion that a patient who moved during the first phase was more likely to move during the second phase as well. Therefore, it is of clinical value to examine the raw data upon completion of the first phase, so that if patient motion is detected additional precautions should be taken in order to avoid motion during the second phase. These usually are based on detailed explanation of the acquisition procedure to the patient (even during periods of great workload) and on close observation of the patient during data acquisition and on reminding the patient that he/she needs to remain motionless. In this regard, the effectiveness of an information pamphlet on reducing motion artifacts during MR imaging [54] and the effectiveness of an ameliorate communication between patient and technologist on reducing patients’ state anxiety scores during PET/CT imaging [55], have been recently demonstrated.

In summary, the link between mood and motion identified in female patients is the first supporting evidence of anxiety as another elementary factor of patient’s cognitive and somatic discomfort during MPI. This is important, since motion is a major cause of falsely positive results in MPI and imaging in general. One could argue that anything that affects the diagnostic accuracy of MPI in women, should be seriously addressed, since many factors, such as smaller heart size, bias of the interpreters, etc., have already been implicated in its lower diagnostic accuracy in this group of patients [56–58].

In our opinion, the identified association between emotional and mental states and motion in women should lead to the development of a suitable questionnaire related patients’ prescan state. Its efficacy as motion predictor should be tested on women in a future study. Identifying patients at risk is helpful as deserve a much closer attention to prevent their motion during the long-lasting imaging modalities in general.

### Limitations

Motion quantification was not performed in our study. However, the careful visual review of projection images is considered the best way to identify any presence of motion [3, 5]. Moreover, the interobserver reliability of more than 90 % in literature [4] and the intraobserver reliability of more than 96 % in our study are in accordance with the high visual motion assessment reproducibility. Visual inspection is a widely acceptable method for detecting vertical body displacements (parallel to the axis of gantry rotation) even in the order of 3.25 mm (corresponding to half pixel in our study) rather than lateral body displacements (perpendicular to the axis of rotation and detected as a fraction of the actual motion in the particular projection). Several reports suggest that vertical motion is the most common type of motion with greater likelihood in producing clinically important artifacts [5, 6, 16].

Another limitation of our study is the acquisition of the projected images by a single-head camera. Multidetector cameras provide a significant advantage over single-detector systems due to decreased image acquisition times (~15 min compared to ~22 min in our study) and, subsequently, a lower risk of patient movement. However, it should be noted that, in case of motion the effect may be compounded, with a single motion being introduced into the dataset 2 or 3 times. Matsumoto and Germano suggest that the exposure to motion for dual gantries should be neutralized to some degree by increasing the time required for single detector acquisition [13, 14].

An issue was the unexpectedly high amount of time required to fill out the psychometric questionnaires. Another issue was the low level of education in women (31.1 %, 14/45), who were primarily from the agricultural region of the country. It could be possible that these women checked “basic education” in the questionnaire, without even having completed the primary education (primary education became compulsory in ‘30s but the law was strictly applied only in ‘70s, when secondary education became compulsory). In that respect, the educational level and the psycho-social background could be the potential factors affecting the high proportion of women who either refused to complete or inaccurately completed the questionnaires. These elderly women might experience a greater insecurity and low self-confidence, hesitating to fill out a questionnaire, since they might be afraid of potential criticism in the event they describe their condition in the questionnaire. Unfortunately, the mode of questionnaire administration, completed by the patient himself as opposed to an interview, could have effect on data quality [59]. So, it may be possible that the routine completion of such questionnaires may interfere with the smooth clinical process and cause substantial delays. An alternative type of simpler questionnaires could be more appropriate for use during clinical practice in an imaging department, which could be distributed and filled out faster and easier.

## Conclusions

The association between anxiety-motion and to a lesser degree of depression-motion identified in female patients represents the first supporting evidence of emotional and mental states as predisposing factors for patient’s motion during MPI. It may be possible that the utilization of appropriate questionnaires prior to the performance of MPI could discriminate more specifically the group of female patients who deserve a much closer attention to prevent their motion.
